# Improvement of an In-Duct Two-Stage Electrostatic Precipitator via Diffusion Charging

**DOI:** 10.3390/toxics10110686

**Published:** 2022-11-12

**Authors:** Ye-Sle Kim, Yeawan Lee, Yong-Jin Kim, Bangwoo Han, Hak-Joon Kim

**Affiliations:** 1 Department of Sustainable Environment Research, Korea Institute of Machinery and Materials, Daejeon 34103, Korea; 2 Environmental & Energy Mechanical Engineering, University of Science and Technology (UST), Daejeon 34113, Korea

**Keywords:** electrostatic precipitator, indoor air, particle charge, particulate matter, diffusion

## Abstract

An in-duct two-stage electrostatic precipitator (ESP) improved by ion diffusion effect was studied. We increased the collection efficiency of a two-stage electrostatic precipitator without additional energy input by adjusting the space arrangement of the charger and the collector, which increased the particle diffusion charging time. The collection efficiency and the particle charge were systematically investigated according to the occurrence of diffusion charging and electric field charging in the charger as generated by the negative ions. The collection efficiency of the separated two-stage ESP was 39% higher, on average, than the theoretical efficiency at the same power consumption. Through simulation, it was verified that the ions generated in the carbon fiber ionizer penetrated the charger. We proposed a modified charging mechanism assuming that the penetrated ions cause additional particle charge. The optimal separation distance between the charger and the collector, which showed the maximum collection efficiency, was derived through the modified charging model. Therefore, the in-duct two-stage ESP developed in this study is a promising energy-efficient and cost-saving design for indoor air management.

## 1. Introduction

Indoor air quality management is an emerging challenge in Asia. In large buildings such as universities, subway stations, and department stores, indoor air quality is controlled by HVAC (heating, ventilation, and air conditioning) systems. According to previous research, an HVAC system may account for more than 50% of the total energy consumption of a building [1,2]. Specifically, Braun et al. (2016) reported that up to 30% of the total energy consumption of an HVAC system may be by the fan [3]. Pressure drop is directly related to the fan power [4]. A filter, which is usually adopted for particulate matter (PM) removal causes an increase in fan energy consumption due to the substantial pressure drop. Although particles can be removed by passing polluted air through the filter, the higher the removal efficiency, the greater the pressure drop [5,6]. Moreover, the filter should be replaced periodically to avoid filter clogging.

An electrostatic precipitator (ESP) removes PM via electrostatic force after charging particles by ions generated through corona discharge. Compared to filters, the ESPs can have a higher PM removal efficiency and a smaller pressure drop, leading to a more energy-efficient air quality management system. However, ESPs currently have limited applicability in indoor air purification because they emit ozone, a toxic gas that causes asthma in children [7,8]; its long-term exposure worsens respiratory and lung function in adults [9]. Further, discharge energy is required for particle charge.

To manufacture lightweight ESPs, a wire-type discharge electrode with a diameter of approximately 100 μm was typically used for commercial ESP cells. However, the wire-type electrode is insufficient to satisfy the World Health Organization ozone concentration guidelines [10]. Poppendieck et al. (2014) evaluated ozone generation by commercial in-duct ESPs in a house. Through the operation of the ESPs, the indoor ozone concentration increased by an average of 58 ppb [11]. To make ESPs more suitable for indoor use, several studies have explored methods for reducing ESP ozone generation. Bo et al. (2010) investigated the corona discharge of carbon nanotubes to minimize the ozone generation [12]. Kim et al. (2016) studied the effect of ionizer material and diameter on the ozone generation [13]. They reported that thin-tipped ionizers discharged a smaller volume of plasma and decreased ozone generation. Therefore, a brush-type ionizer, which has a diameter of less than 10 μm, was used to reduce ozone emissions while maintaining the ion generation of a two-stage ESP. Shin et al. (2019) evaluated the ozone generation and particle collection efficiency of wire electrodes, aluminum foil, and carbon fabric [14]. The carbon fabric exhibited superior performance with minimum ozone generation compared to the others. Katatani et al. (2016) and Takashima et al. (2018) studied two-stage ESPs using induction charging without corona discharge to prohibit ozone emission [15,16].

Although the ozone emission can be reduced by carbon brush ionizers, the reduced discharge energy decreases collection efficiency. According to the conventional ESP theory, the particle charge, collection area, and electric field strength should be increased to improve collection efficiency [17,18]. To increase the collection area, either the distance between the collection plates has to be narrowed or the size of the collection plates must be increased [19]. To increase the electric field strength, a high voltage should be applied. All of the above methods directly increase operation costs.

For the building HVAC, indoor polluted air is ventilated by the air handling unit, and clean air is supplied back into the building [20]. This process is repeated and therefore the interior of the building can be considered as an expanded semi-closed chamber. Conventional two-stage ESPs treat the charger and collector as a set and are situated close to each other for scalability. However, within the structure of an HVAC system operating in a semi-closed chamber, the charger and collector do not need to be in proximity. In fact, each part of a two-stage ESP can be positioned anywhere within the ventilation duct. Thus, the contact time of particles and ions generated by the charger can be prolonged, and particle charge rates can be increased by the diffusion charging effect.

In this study, we increased the distance between the charger and the collector of an in-duct two-stage ESP to improve collection efficiency via diffusion. To the best of our knowledge, no study has been conducted on the effect of distance between the charger and collector on the performance of two-stage ESPs. We fabricated a two-stage ESP that consisted of a charger with carbon brush ionizers and a collector with carbon films to reduce ozone.

## 2. Materials and Methods

**Figure 1:**
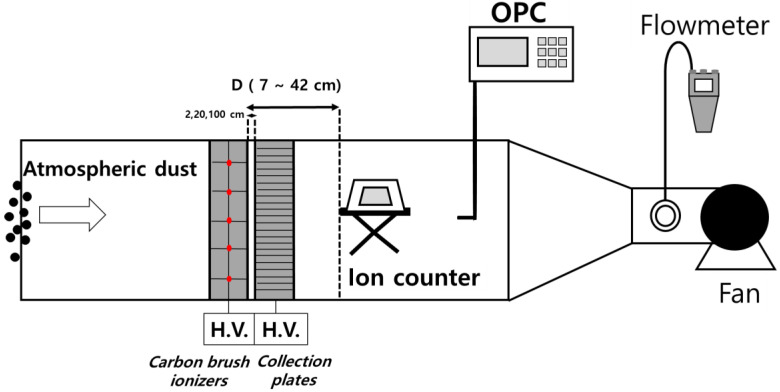
Schematic of collection efficiency measurement experiment.

In this study, the particle collection efficiency of the two-stage ESP and the number of ions from the charger were measured. Figure 1 is a schematic of the experiment. The test duct had an area of 0.6 m × 0.6 m, and the flow velocity inside the duct was measured with a vane-type flow meter (TESTO 480, Testo SE & Co. KGaA, Lenzkirch, Germany). The flow rate was measured in a circular duct (200 mm diameter) downstream of the particle sampling probe. In all experiments in this study, the flow rate was fixed at 43.2 m^3^/min, and the flow velocity was 2 m/s. The duct that the air flowed through was grounded to exclude the external electric field effect.

In the experiment for measuring particle collection efficiency, the distance between the charger and collector was 2 cm, which was the width of the case of the collector. The distance between the charger and the particle sampling probe was 50 cm. The test particles consisted of atmospheric dust introduced into the room. The applied voltages on the charger were −12, −13, −14, and −15 kV, and their power consumptions were 3.78, 5.07, 6.58, and 8.48 W, respectively. For each applied charger voltage, measurements of −8 and −9 kV were obtained for two collector voltages, respectively. Particle concentration downstream of the ESP was measured with an aerosol spectrometer (Model 1.109, Grimm, Ainring, Germany). The particle collection efficiency was calculated by the following Equation (1).

(1) $$\eta \left(\%\right)=\left(1-\frac{n_{2}}{n_{1}}\right)\times 100$$

where *η* is the particle collection efficiency (%), *n*_1_ is the particle number concentration before operating ESP (particles/m^3^), and *n*_2_ is particle number concentration after operating ESP (particles/m^3^). We measured the particle at a diameter of 0.3 μm to estimate collection efficiency. All particle collection efficiency measurements were performed in triplicate. For each experiment, *n*_1_ and *n*_2_ were measured at intervals of 6 s for 10 points during 1 min and 30 s, and the average value was used when calculating collection efficiency. For the experimental error, the standard deviation between the triplicate experiments was calculated and marked as an error bar. The initial particle concentration was 9 ± 1 × 10^7^ particles/m^3^. The variation in atmospheric particle concentration during the experiment was approximately 5 × 10^6^ particles/m^3^, which was within 10% of the initial concentration. The proportion of naturally charged particles among the initial number of particles was less than 5%. Naturally charged particles were measured by calculating the collection efficiency of the collector. At this time, collection efficiency was calculated by Equation (1) operating only the collector without the charger.

Ozone concentration was measured using an ozone analyzer (O3 analyzer T400, Teledyne Technologies, Thousand Oaks, CA, USA) at the same sampling location as the aerosol spectrometer. The concentration of generated ozone was defined as the increased ozone concentration after operating ESP compared to the background ozone concentration. For measuring the ion concentrations, we placed an ion counter (IM806V2, Umweltanalytik Holbach GmbH, Wadern, Germany) downstream of the charger, wherein the flow rate was 43.2 m^3^/min. The ionometer measures air ions with an electrical mobility of 1.3 cm^2^/V∙s and faster. In the ion concentration measurement experiment, only the charger and ion counter were positioned in the test duct. The distance between the charger and the ion counter ranged between 7 and 42 cm to examine how the ion concentration decreased after penetrating the charger.

**Figure 2:**
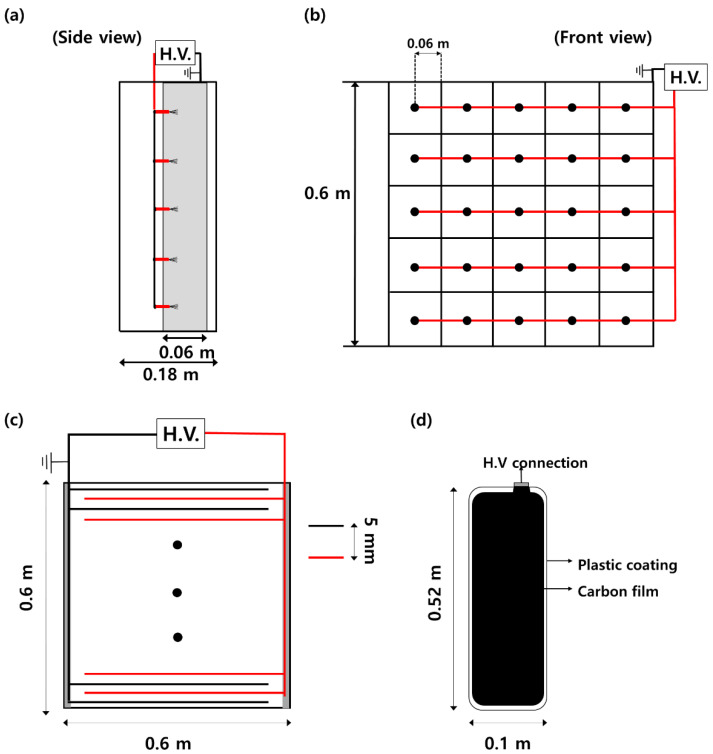
Schematic of the charger and collector of the two-stage electrostatic precipitator: (**a**) Side view of the charger; (**b**) Front view of the charger; (**c**) Front view of the collector; (**d**) High-voltage electrode of the collector (carbon film coated with plastic).

In this study, the two-stage ESP consisted of a charger with carbon fiber ionizers and a parallel plate-type collector (Figure 2). Figure 2a,b depict the side and front views of the charger, respectively. The charger with carbon fiber ionizers (0.6 [W] × 0.18 [L] × 0.6 [H] m) had a cross-sectional area of 0.36 m^2^. The carbon fiber discharge electrodes were placed in the center of a square ground plate in a 5 × 5 channel. The width of the aluminum ground plate was 6 cm. The distance between the discharge electrode and the ground plate was 6 cm.

Figure 2c shows the collector (0.6 [W] × 0.11 [L] × 0.6 [H] m) composed of plate-type high-voltage electrodes and ground electrodes. The high-voltage electrode was a carbon film coated with plastic (Figure 2d), and the aluminum ground electrode had the same size as the carbon film. Notably, 50 high-voltage plates and 51 ground plates were arranged at intervals of 5 mm and the collection area was 5.2 m^2^.

## 3. Results and Discussion

### 3.1. Characterization of the ESP Performance

**Figure 3:**
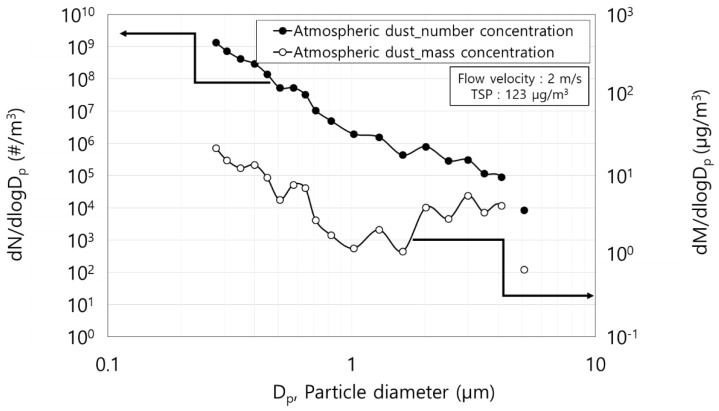
Particle size distribution of the atmospheric dust.

Figure 3 shows the particle size distribution of the atmospheric dust tested in this study. The total of suspended particles (TSP) was 123 μg/m^3^. The number and mass concentration of the 0.27 μm particles were highest among all the particles at 1.4 × 10^9^ particles/m^3^ and 22 μg/m^3^, respectively. In a study by Ji (2020), the highest mass concentration of dust floating in the room was below 1 μm [21]. In this study, the particle size distribution of atmospheric dust in the laboratory also showed the same trend. The particles of size 0.3 μm, which are the object in this study, accounted for 81% and 41% of the total particle number and mass concentration, respectively.

**Figure 4:**
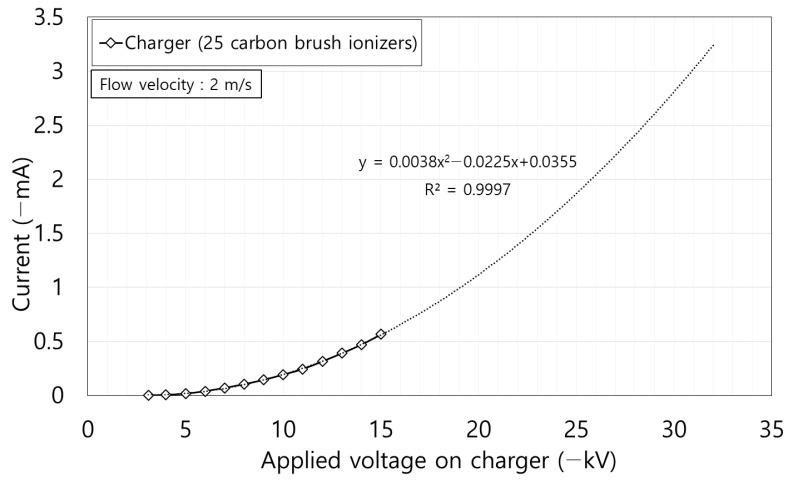
Corona discharge current according to the applied voltage of the charger.

Figure 4 shows the corona discharge current according to the voltage applied in the charger consisting of 25 carbon fiber ionizers. Corona discharge started when −3.1 kV was applied to the charger and the current was 0.565 mA at the maximum applied voltage of −15 kV. According to Townsend’s law, the corona discharge current curve, with respect to the applied voltage, has a quadratic form [22]. The corona discharge current curve in this study also showed a quadratic tendency and the coefficient of determination (R^2^) was 0.9997 and was judged to confer a high prediction accuracy to the trend line.

**Figure 5:**
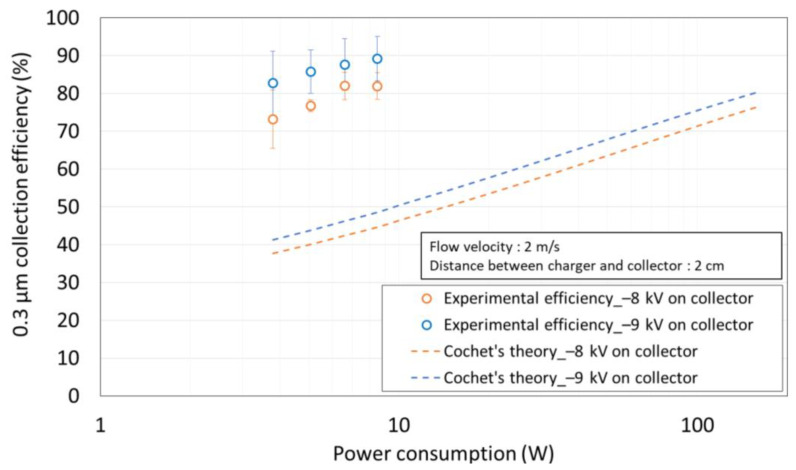
Comparison of the experimental efficiency and Cochet’s theoretical efficiency according to the power consumption.

Figure 5 shows the comparison of the experimental efficiency of the two-stage ESP in this study and Cochet’s theoretical efficiency according to power consumption. The variation in atmospheric particle concentration was within 8% of the initial concentration. The maximum particle collection efficiency was 89.2% at the maximum power consumption of the charger (8.48 W) and the maximum applied voltage of the collector (−9 kV). The particle collection efficiency increased as the power consumption of the charger increased due to the enhanced particle charge.

Theoretically, the collection efficiency of an ESP follows Equations (2) and (3) [23].

(2) $$\eta =1-\exp\left(-\frac{W_{m}A}{Q}\right)$$

(3) $$W_{m}=\frac{Q_{p}E_{c}C_{c}}{{3\pi \mu d}_{p}}$$

where, *η* is the theoretical particle removal efficiency of the ESP (%), *Q* is the flow rate (m^3^/s), *W_m_* is the migration velocity (m/s), *A* is the collection area (m^2^), *Q_p_* is the particle charge (C), *E_c_* is the electric field strength of the collector (V/m), C_c_ is Cunningham correction factor (1.574 for a particle in a diameter of 0.3 μm), *μ* is the dynamic viscosity of air (1.8 × 10^−5^ Pa·s), and *d_p_* is the particle diameter (m).

Additionally, due to the mechanism of particle charging by corona discharge [23], the amount of charge on the particles increases as the electric field strength and charge time of the particles increase. Equation (4) is the diffusion charge of the particle and Equation (5) is the electric field charge of the particle [24,25].

(4) $$n{\left(t\right)}_{,diff}=\left(\frac{d_{p}kT}{2K_{E}e^{2}}\right)\ln\left[1+\frac{\pi K_{E}d_{p}c_{i}e^{2}N_{i}t}{2kT}\right]$$

(5) $$n{\left(t\right)}_{,field}=\left(\frac{3\varepsilon_{r}}{\varepsilon_{r}+2}\right)\left(\frac{E_{c}d_{p}{}^{2}}{4K_{E}e}\right)\left(\frac{{\pi K}_{E}eZ_{i}N_{i}t}{1+{\pi K}_{E}eZ_{i}N_{i}t}\right)$$

where *n*(*t*) is the number of charges, *d_p_* is the particle diameter (m), *t* is the charging time (sec), *c_i_* is the mean thermal speed of the ions (240 m/s), *N_i_* is the concentration of ions (#/m^3^), *k* is the Boltzmann constant (J/K), *T* is temperature (K), *e* is the charge of an electron (1.6 × 10^−19^ C), *K_E_* is the electrostatic constant of proportionality (9 × 10^9^ N∙m^2^/C^2^), *ε_r_* is the relative permittivity (3.2), *E_c_* is the electric field strength of charger (V/m), and *Z_i_* is the mobility of ions (1.5 × 10^−4^ m^2^/V∙s).

The theoretical efficiency was obtained using Equations (2) and (3). To estimate the particle charge, Cochet’s charging theory in Equation (6) was used.

(6) $$Q_{p}{}^{\infty }=\left\{{\left(1+\frac{2\lambda }{d_{p}}\right)}^{2}+\left(\frac{2}{1+2\lambda /d_{p}}\right)\left(\frac{\varepsilon_{r}-1}{\varepsilon_{r}+2}\right)\right\}\times {\pi \varepsilon }_{0}d_{p}{}^{2}E_{p}$$

$Q_{p}^{\infty }$ is particle charge (C), *d_p_* is diameter of particle (m), *λ* is mean free path (m), and *ε_r_* is electrical permittivity. For most materials, 1 < *ε_r_* < 10 [23], *ε_r_* is 3.2 for diesel soot [26,27], 4.3 for quartz, 3.9 for SiO_2_, and 5 < *ε_r_* < 9 for mineral dust [28,29,30]. In this study, we set *ε_r_* as 3.2, because ultrafine particles less than 1 μm are typically originated from chemical production such as diesel soot. ε_0_ is vacuum permittivity (8.9 × 10^−12^ F/m), and *E_p_* is electric field of charger (V/m). Using the corona discharge current predicted from the graph in Figure 5 based on the applied voltage at the charger, the theoretical efficiency according to power consumption was up to 163 W.

As a result, the experimental efficiencies were on average 1.9 times higher than the theoretical efficiencies at the same power consumption. Additionally, when the applied voltage on the collector was −9 kV, the experimental results showed a particle collection efficiency of 83% at 3.78 W, while the theoretical efficiency was 81% at 163 W. Therefore, it was confirmed that, theoretically, the power consumption required for achieving a similar particle collection efficiency as obtained in this experiment was 43 times or more than that used in this experiment.

### 3.2. Development of the Modified Charging Model

**Figure 6:**
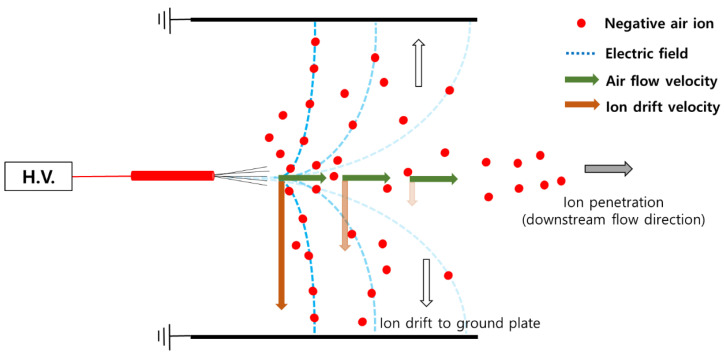
Movement path of negative ions generated inside the ionizing channel used.

We speculated that the reason for the experimental efficiency being considerably higher than the theoretical prediction shown in Figure 5 was the effect of diffusion charging by the negative air ions, which penetrated the charger. Figure 6 shows the movement path of negative ions generated inside the ionizing channel used in this study. The high voltage applied to the carbon fiber ionizes the air by generating a strong electric field locally. Negative air ions pass along the air flow and at the same time drift to the ground electrode by an electric field formed in the ionizing channel. While the air flow rate is constant, the electric field in the ionizing channel decreases when farther from the carbon fiber, i.e., the ion drift velocity decreases as the distance from the carbon fiber increases. Therefore, at a short distance from the carbon fiber, most of the negative ions move to the ground electrode by ion mobility, and beyond a certain distance, the effect of the electric field becomes weaker compared to the air flow. Then, the negative ions pass through the ionizing channel and move to the collection stage.

**Figure 7:**
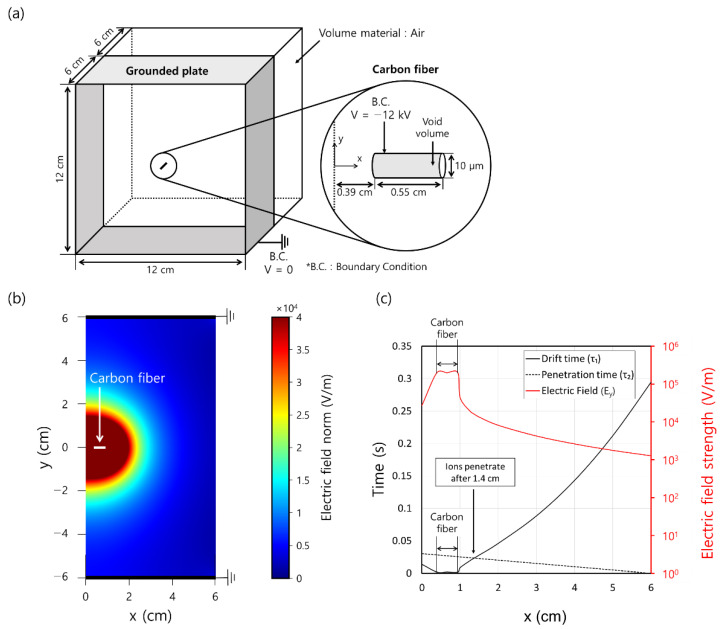
(**a**) Configuration of an ionizing channel used in COMSOL simulation. (**b**) Electric field distribution of a cross-section of the *x*-*y* plane of an ionizing channel. (**c**) Ion drift time, ion penetration time, and average electric field strength of the *y*-direction according to *x*.

To study the spatial distribution of the electric field inside the ionizing channel, the finite element method was used to solve the model equations using COMSOL Multiphysics software (COMSOL, Inc., Stockholm, Sweden). The computational procedure of COMSOL simulation is as follows: establishing geometric entity, defining material property, building physics model (Electrostatics, Laminar flow, etc.), designating boundary conditions, constructing mesh, solving, and post-processing. Herein, we used only the electrostatics physical model for the study. In a steady state, charge conservation equations according to Gauss’ law were as follows:

(7) $$\vec{E}=-\nabla V$$

(8) $$\nabla \cdot \left(\varepsilon_{0}\varepsilon_{r}\vec{E}\right){=\rho }_{v}$$

where $\vec{E}$ is the electric field vector, *V* is electric potential, *ε*_0_ is the vacuum permittivity, *ε*_*r*_ is the relative permittivity, and *ρ_v_* is the space charge density. For simplicity, we analyzed only a thin cylindrical fiber (10 μm diameter and 5.5 mm length). The specific geometry and boundary condition of the single ionizing channel are illustrated in Figure 7a. The number of mesh was 747,470, and the minimum skewness was 0.5. Vacuum permittivity and relative permittivity of air was set as 8.85 × 10^−12^ C/(m∙V) and 1, respectively.

Figure 7b shows the electric field distribution on a cross-section lying on the *x*-*y* plane of an ionizing channel, as obtained using COMSOL. The white line indicates the carbon fiber and the bold black lines at the top and bottom indicate the ground plate. −12 kV was set to the carbon fiber. As we mentioned earlier, the electric field strength decreases rapidly as the distance from the carbon fiber increases. From the numerical results, the drift time *τ*_₁_, which is the time required for ions to reach the ground plate, can be defined as follows:

(9) $$\tau_{1}=\frac{d}{Z_{i}E_{y}}$$

where *d* is the distance between carbon fiber and ground plate (6 cm), *Z_i_* is the negative ion mobility (0.00015 m^2^/V·s) [23], *E*_y_ is the average electric field strength of *y*-direction from *y* = 0 to *y* = 6 cm. From the air flow, the penetration time *τ*_2_, which is the time required for ions to penetrate the ionizing channel, was defined as:

(10) $$\tau_{2}=\frac{l\;-\;x}{U}$$

where *l* is the channel length (6 cm), *U* is the flow velocity. Here, we assumed that ions are at the *y* = 0 line for simplicity.

Figure 7c shows the ion drift time, ion penetration time, and average electric field strength in the *y*-direction as a function of *x*. From *x* = 0.39 cm to *x* = 0.94 cm, where the carbon fiber is located, the average electric field strength is 2 × 10^5^ V/m, which is the same as that when the charger voltage is −12 kV and electrode spacing is 6 cm. After point *x* = 0.94 cm, which is at the edge of the carbon fiber, the electric field strength decreases rapidly to 1.3 × 10^5^ V/m. Due to the decreased electric field strength, the ion drift time increases rapidly. However, the ion penetration time decreases gently in the form of a linear function from 0.03 s to 0. From the graph, it is clear that the ions drift to the ground plate due to ion mobility before *x* = 1.4 cm. After *x* = 1.4 cm, the penetration time becomes shorter than the drift time (*τ*_₁_ > *τ*_2_), which implies that the ions passed through the ionizing channel and moved along the duct.

**Figure 8:**
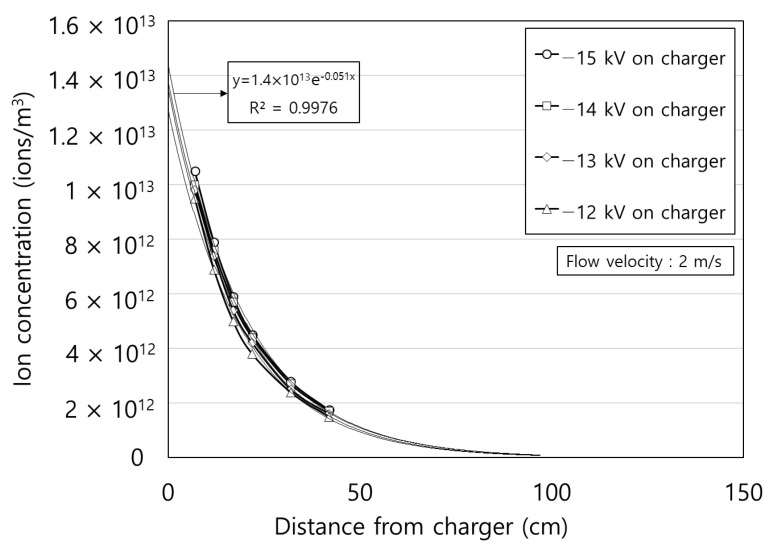
Ion concentration according to the distance from the charger.

Figure 8 shows the ion concentration according to the distance from the charger. The applied voltages on the charger were −12, −13, −14, and −15 kV. The ion concentrations decreased exponentially with distance and slightly increased as the applied voltage increased. For the exponential fitted line for the four experimental results, the coefficients of determination, indicated by the R^2^ values, are all over 0.99, which is consistent with the results of previous studies [31,32,33]. This can be explained by the ion convection-diffusion equation as below:

(11) $$\frac{\partial N_{i}}{\partial t}+\left(\vec{u}+Z_{i}\vec{E}\right)\cdot \nabla N_{i}=D_{p}\nabla^{2}N_{i}$$

where *N_i_* is the ion concentration, $\vec{u}$ is the air flow vector, *D_p_* is the diffusion coefficient of air ion. The electric field in the duct originated from the negative ions. Here, we assumed that the ions are well dispersed in the duct. All the experiments were performed in a steady state. Therefore, the diffusion of negative air ion and the time-dependent term can be negligible leading to the below equation.

(12) $$\left(\vec{u}+Z_{i}\vec{E}\right)\cdot \nabla N_{i}=0$$

From the divergence theorem, volumetric integration of Equation (13) can be expressed as follows:

(13) $$∯\left(N_{i}\vec{u}\right)\cdot \hat{n}dS+∯\left(Z_{i}N_{i}\vec{E}\right)\cdot \hat{n}dS=0$$

where $\hat{n}$ is the outward pointing unit normal vector at each point on the boundary of the control surface *S*. The coordinate system is the same as that shown in Figure 8. According to [34], the mathematical solution of Equation (13) becomes a summation of a series of exponential terms. From the second term onwards, there is no significant effect on the result. The first term of ion concentration is expressed as an exponential function of *x* as follows:

(14) $$N_{i}(x)={\;N}_{0}\times \exp\left(-\frac{k\;x}{Q}\right)$$

where *k* is the decay constant due to the electric force in m^2^/s, *N*_0_ is the ion concentration at *x* = 0, and *Q* is the air flow rate.

**Figure 9:**
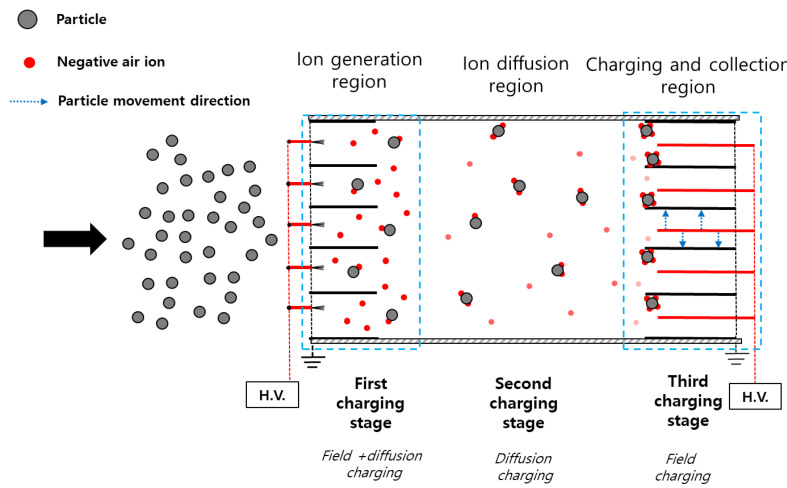
Schematic of the modified charging mechanism of the two-stage electrostatic precipitator using carbon fiber ionizers with the charger and collector separated.

Figure 9 shows a schematic of the particle charging mechanism of the two-stage ESP in this study. The charger with the carbon fiber ionizer was distant from the collector. We developed a modified charging model based on the concept of multiple charging via three stages. In the 1st stage, where negative ions are generated, diffusion charging and electric field charging occur in the charger following Cochet’s charging mechanism. We concluded that Cochet’s charging mechanism is adequate for this experiment rather than the dynamic model including the local field effect because according to [35], for typical electrical states, the saturation charging time was less than 10 ms, which was shorter than the particle penetration time in the 1st stage. In addition, for the application of the dynamic charging model, the ion concentration inside the charger is required. However, it was almost impracticable to measure the ion concentration generated by the ionizer in the charger without electrical interference due to the confined geometry of the ionizing channel.

In the 2nd stage, there is an ion diffusion region. In this stage, we ruled out the coagulation effect [23]. The coagulation of aerosols results in a continuous decrease in number concentration coupled with an increase in particle size. The number concentration at a certain time can be calculated as follows.

(15) $$N\left(t\right)=\frac{N_{p,0}}{1+N_{p,o}Kt}$$

where *N(t)* is the number concentration at time *t* (#/m^3^), *N_p,_*_0_ is the original number concentration at time zero (#/m^3^), *t* is coagulation occur time (s). *K*, the corrected coagulation coefficient (m^3^/s) can be calculated as follows.

(16) $$K=3.0\times 10^{-16}C_{c}\beta$$

where *C_c_* is the Cunningham correction factor, *β* is the correction factor of dynamic shape (0.93 for a particle in a diameter of 0.3 μm). During the maximum penetration time in this study (0.5 s), the number concentration decreases by a factor of 1 under the experiment conditions, which means there is no coagulation effect of particles. The experiment conditions are the flow velocity of 2 m/s, maximum initial concentration of 10^8^ #/m^3^, particle diameter of 0.3 μm, maximum penetration length of 1 m and the corrected coagulation coefficient of 4.34 × 10^−16^ m^3^/s. Diffusion charging occurs due to the penetrated negative ions in the section between the charger and the collector where no electric field exists. In the 3rd stage, there is an electric field charging and particle collecting region. In this stage, the ions that passed through the 2nd stage are subjected to electric field charging in the collector, and then the charged particles are removed by electrostatic force.

In the modified charging mechanism, it is assumed that the total charge number is the sum of all charge numbers generated in three stages. In this case, the total charge number may exceed the charge limit of Equation (5), where time *t* approaches infinity, because the particles are not exposed to a continuous strong electric field. According to [23], where there is no strong external field, a much higher charge than the maximum charge in field charging can be achieved before the limit of spontaneous charge loss is reached. In the modified charging mechanism, there is no strong external electrical in the 2nd stage and the charged particles pass through the 3rd stage before achieving the charge limit. The charge limit where the self-generated field at the surface of a particle reaches the value required for spontaneous emission of electrons from a surface can be calculated as follows:

(17) $$n_{L}=\frac{d_{p}{}^{2}E_{L}}{4K_{E}e}$$

where *d_p_* is the particle diameter (m), *K_E_* is the electrostatic constant of proportionality (9 × 10^9^ N∙m^2^/C^2^), *E_L_* is the surface field strength required for spontaneous emission of electrons (9.0 × 10^4^ V/m), *e* is the charge of an electron (1.6 × 10^−19^ C). For a particle with a diameter of 0.3 μm, the maximum charge is 14,062.

To apply the particle charging mechanism shown in Figure 9, we modified Equations (4) and (5), which are based on the conventional charging theory of particles by corona discharge [23]. In the modified charging model of this study, we increased the charge of the particle in sequential steps. The equations for the particle charging are as follows:

(18) $$\Delta n{\left(x_{j}\right)}_{,diff}=\left(\frac{d_{p}kT}{2K_{E}e^{2}}\right)ln\left(1+\frac{\pi K_{E}d_{p}c_{i}e^{2}N_{i}\left(x_{j}\right)\Delta t_{d}}{2kT}\right),\;j=1,2,\cdots ,N$$

(19) $$n{\left(x_{j}\right)}_{,diff}=n{\left(x_{0}\right)}_{,diff}+\overset{k=j}{\underset{k=1}{\sum }}\Delta n\left(x_{k}\right)\;$$

(20) $$x_{j+1}=x_{j}+\Delta x$$

(21) $$\Delta t_{d}=\Delta x/U$$

(22) $$x_{0}=0,\;\;\;\;x_{N}=d$$

(23) $$n{\left(x_{0}\right)}_{,diff}=0$$

(24) $$n{\left(x_{N}\right)}_{,field}=\left(\frac{3\varepsilon_{r}}{\varepsilon_{r}+2}\right)\left(\frac{E_{c}d_{p}{}^{2}}{4K_{E}e}\right)\left(\frac{{\pi K}_{E}eZ_{i}N_{i}\left(x_{N}\right)t_{f}}{1+{\pi K}_{E}eZ_{i}N_{i}\left(x_{N}\right)t_{f}}\right)$$

where *x* is the distance from the charger, △*x* is the step size of *x_j_*, *n(x_j_)_,diff_* is the particle charge number by diffusion charging in a position of *x = x_j_, n(x_N_)_,field_* is the particle charge number by electric field charging in the 3rd stage, *N_i_(x_j_)* is ion concentration at a distance of *x_j_* from the charger (#/m^3^), △*t_d_* is particle residence time as it moves by △*x* (sec), *E_c_* is the electric field of collector, *U* is the flow velocity (m/s), *d* is the distance between the charger and the collector (m), and *t_f_* is the residence time of the particle in the 3rd stage. The definitions of the other variables are the same as those in Equations (4) and (5). *U* is 2 m/s, and we set △*x* and *t_f_* as 1 cm and 0.05 s, respectively.

**Figure 10:**
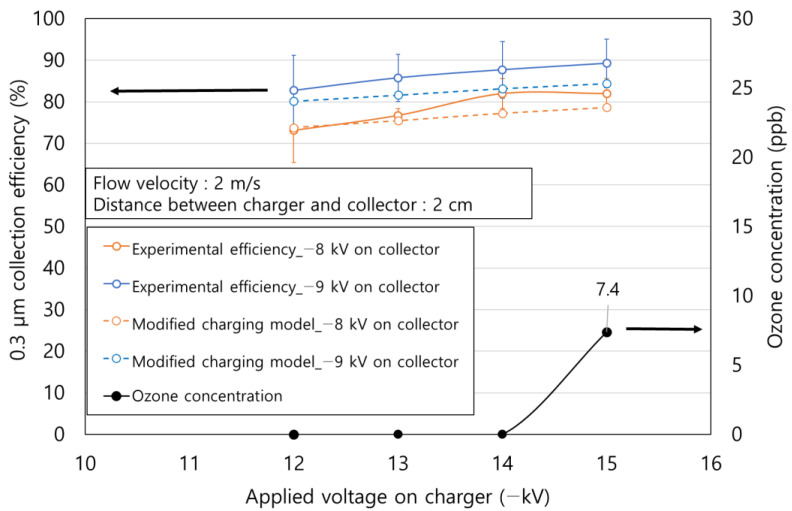
Comparison of the experimental efficiency and modified charging model according to the applied voltages.

The theoretical efficiency of the two-stage ESP with the modified charging model was compared with the experimental efficiency (Figure 10). In the experiment, the charger and collector were 2 cm apart. As a result, under all applied voltage conditions, the experimental efficiency and the theoretical efficiency of the modified charging model showed a difference of 3.3% on average. Therefore, when the ions penetrated the charger, we infer that the mechanism of the modified charging model was followed rather than the conventional particle charging model of Cochet [17]. The maximum ozone concentration of the carbon brush ionizer was 7.4 ppb, and not generated below the maximum test voltage of −15 kV.

### 3.3. Effect of the Distance between Charger and Collector

**Figure 11:**
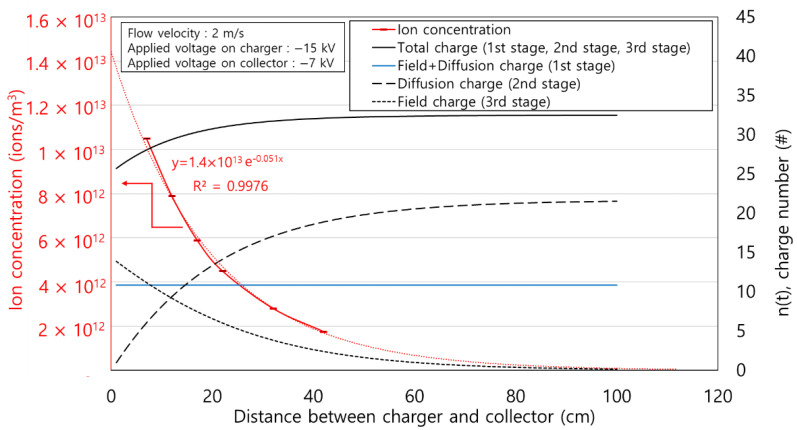
Obtained particle charge number at each stage according to the distance between the charger and collector using the modified charging model.

Figure 11 shows the particle charge number at each stage using the modified charging model in this study and ion concentration according to the distance between the charger and the collector, *d* (cm). The voltage applied to the charger and the collector was −15 kV and −7 kV, respectively. The particle charge number of the 1st stage was obtained using Equation (6). In the 1st stage, the particle charge number was constant because of the fixed applied voltage on the charger (−15 kV). Using Equations (18) and (24), it was confirmed that the diffusion charge number of the 2nd stage increased, and the electric field charge number of the 3rd stage decreased as *d* (cm) increased. This is owing to the ions generated in the ionizer that pass through the charger. As the *d* (cm) increased, the diffusion charging time increased, and an increase was observed in the 2nd stage particle charge number. Contrarily, after ions penetrated the charger, the passed ions decreased exponentially according to *d* (cm), as shown in Figure 8. The electric field charge number of the 3rd stage decreased as the *d* (cm) increased. The total charge number is the sum of all charge numbers generated from 1st to 3rd stage. The total charge number increased as the *d* (cm) increased.

**Figure 12:**
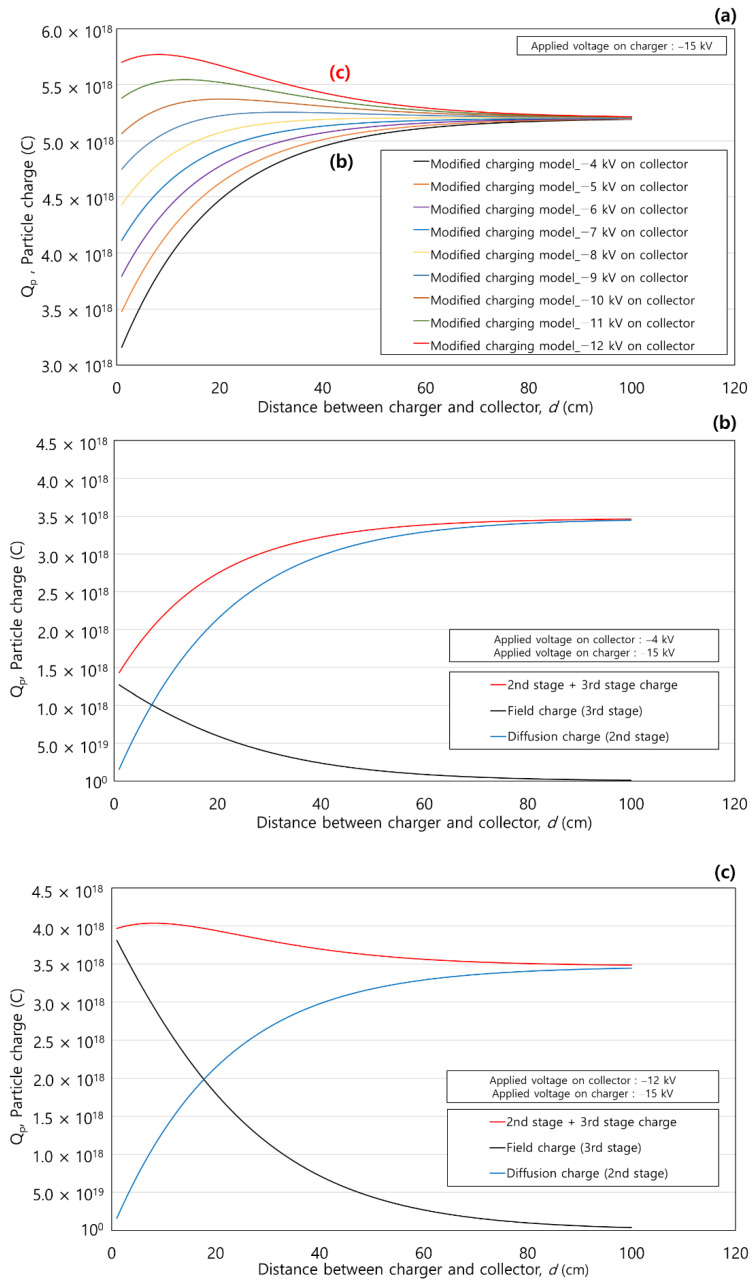
Comparison of the particle charge variances according to the distance between the charger and the collector using the modified charging model: (**a**) Total particle charge according to the applied voltage on the collector (−4 kV to −12 kV); (**b**) Particle charge of the 2nd and 3rd stages with −4 kV on the collector; (**c**) Particle charge of the 2nd and 3rd stages with −12 kV on the collector.

Figure 12a shows the comparison of the total particle charge variances according to the *d* (cm) using the modified charging model with various applied voltages of the collector. The applied voltage of the charger was fixed at −15 kV and the applied voltages on the collector were −4 to −12 kV. Contrary to the results shown in Figure 11, the increasing or decreasing trends of particle charge correlated with changes in the applied voltage on the collector. When the applied voltage to the collector was low (−4 to −9 kV), the total charge tended to continuously increase as *d* (cm) increased. Whereas, when the applied voltage to the collector was high (−10 to −12 kV), the total charge initially increased and then decreased after reaching the maximum value at a specific *d* (cm). When *d =* 100 cm and the ion concentration converged to 0, the total charge converged to a constant value (5.2 × 10^−18^ C) under all applied voltages.

Figure 12b,c show particle charge variances according to *d* (cm) when the applied voltages on the collector were −4 kV and −12 kV, which are the minimum and maximum voltages, respectively [Figure 12a]. The particle charge of the 1st stage was not indicated because it was constant when the applied voltage on the charger was fixed. Figure 12b,c show the same diffusion charge as the 2nd stage according to *d* (cm) because the penetrated ion concentration is the same. In both figures, the trends for the 2nd and 3rd stages are the same. However, the increasing or decreasing trend of total particle charge was changed by the balance of the charging at the 2nd and 3rd stages. For instance, in Figure 12b, the total charge increased with *d* (cm) because the field charging in the 3rd stage was much weaker than the diffusion charging in the 2nd stage owing to the low voltage of the collector (−4 kV). In this case, the maximum value of 3.5 × 10^−18^ C was achieved at *d =* 100 cm. As shown in Figure 12c, when a voltage of −12 kV was applied to the collector, the total charge initially increased and then decreased after achieving the maximum value of 4 × 10^−18^ C at *d* = 9 cm because the field charging in 3rd stage was dominant.

**Figure 13:**
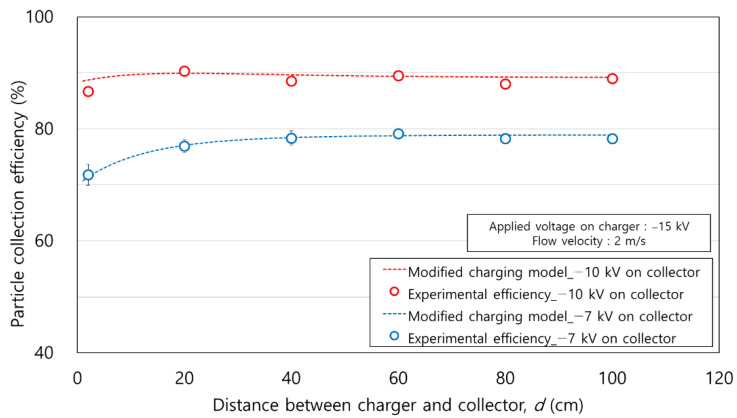
Comparison of the experimental efficiency and modified charging model according to the distance between charger and collector.

Figure 13 shows a comparison of the experimental efficiency and modified charging model according to *d* (cm) and applied voltage to the collector. In the experiment, the applied voltage to the charger was fixed at −15 kV, and the applied voltages to the collector were −10 kV and −7 kV. The experiments were conducted at *d =* 2–100 cm. The variation in atmospheric particle concentration was within 3% of the initial concentration. Compared to the experiment shown in Figure 5, a relatively uniform particle concentration was observed in the experiment in Figure 13. The maximum particle collection efficiency estimated by the modified charging model was 78.9% at *d =* 100 cm when the applied voltage on the collector was −7 kV. At an applied voltage of −10 kV on the collector, the maximum particle collection efficiency estimated by the modified charging model was 89.9% at *d* = 20 cm. Under all conditions of *d* (cm), the differences in experimental efficiencies and the modified charging model were 0.3% and 0.8% on average with −7 kV and −10 kV on the collector, respectively. As a result, the increasing or decreasing trend of particle charge according to the applied voltage and *d* (cm) in the modified charging model was experimentally verified.

## 4. Conclusions

In this study, we widened the distance between the charger and the collector of the two-stage ESP. We increased the diffusion charging time of particles by negative ions that penetrated the charger. We studied the characteristics of collection efficiency and particle charge variations according to the separation distance. The separated two-stage ESP showed higher collection efficiency of 39% on average than the theoretical ESP at the same power consumption. Therefore, a modified charging model to explain the improvement of collection efficiency was suggested, consisting of three stages.

(1) The 1st stage is the ion generation region, where diffusion and electric field charging occur in the charger following Cochet’s charging mechanism.
(2) The 2nd stage is the ion diffusion region, where diffusion charging occurs due to the penetrated negative ions in the section between the charger and collector.
(3) The 3rd stage is the electric field charging and particle collecting region, where the ions that passed through the 2nd stage are subjected to electric field charging in the collector, and the charged particles are then removed by an electrostatic force.

The modified charging model presented in this study was consistent with the experimental collection efficiencies with a difference of less than 5%. Moreover, the modified charging model indicated that there was an optimal separation distance between the charger and collector for achieving maximum collection efficiency, which was verified experimentally.

The separated two-stage ESP can be used to improve indoor air quality in buildings that use air-handling units, such as universities, subway stations, and department stores. It is suitable for indoor purification with high collection efficiency and low ozone generation using carbon fiber ionizers. Additionally, the collection efficiency of the separated two-stage ESP can be increased by changing the structure of the ESP system without additional power consumption. Therefore, the separated two-stage ESP developed in this study is economical and applicable to indoor air conditioning systems with a novel modified charging model. The modified charging model is expected to be applied to future studies on two-stage ESPs.

## Data Availability

Not applicable.
